# Alpha-foetoprotein in umbilical cord in relation to severe pre-eclampsia, birth weight and future breast cancer risk

**DOI:** 10.1038/sj.bjc.6600125

**Published:** 2002-03-04

**Authors:** L J Vatten, P R Romundstad, R A Ødegård, S T Nilsen, D Trichopoulos, R Austgulen

**Affiliations:** Institute of Community Medicine and General Practice, University Medical Centre, Norwegian University of Science and Technology, Trondheim, Norway; Institute of Cancer Research and Molecular Biology, Norwegian University of Science and Technology, Trondheim, Norway; Department of Obstetrics and Gynecology, Rogaland Central Hospital, Stavanger, Norway; Department of Epidemiology and Center for Cancer Prevention, Harvard School of Public Health, Boston MA, USA

**Keywords:** pre-eclampsia, oestradiol, α-foetoprotein, birth weight, breast cancer

## Abstract

Women born after pre-eclamptic pregnancies have been reported to be at reduced risk of breast cancer as adults, because of reduced intrauterine oestrogen influence on breast tissue; high levels of α-foetoprotein (a glycoprotein with anti-oestrogenic properties), however, could also be important. In severe pre-eclampsia, placental function and foetal growth are reduced, and umbilical cord plasma levels of α-foetoprotein could reflect the underlying processes. Umbilical cord blood was collected in 12 804 consecutive deliveries. Among 307 pregnancies with clinical pre-eclampsia, 66 singleton pregnancies were identified as clinically severe, and 610 singleton pregnancies were selected as controls. Oestradiol and α-foetoprotein were measured from umbilical plasma, and birth weight was standardized as the ratio between the observed and expected birth weight, adjusted for differences in gestation length and offspring sex. Cord plasma levels of α-foetoprotein were significantly higher in severe pre-eclampsia than controls (*P*<0.01) after adjustment for gestational age and birth weight. For oestradiol, there was no difference in cord plasma levels between the severe pre-eclampsia group and controls, after adjustment for length of gestation and birth weight. These results suggest that an anti-oestrogenic effect associated with pre-eclampsia may be mediated through high levels of α-foetoprotein rather than low levels of oestradiol.

*British Journal of Cancer* (2002) **86**, 728–731. DOI: 10.1038/sj/bjc/6600125 www.bjcancer.com

© 2002 Cancer Research UK

## 

The hypothesis that breast cancer may originate *in utero* implies that intrauterine factors that lead to higher birth weight increase the risk of breast cancer in female offspring (Trichopoulos, 1990; Anbazhagan and Gusterson, 1994). The evidence related to birth weight is suggestive, but not conclusive (Potischman and Troisi, 1999). Two large Swedish studies, however, have shown that women born after pre-eclamptic pregnancies have a risk reduction for breast cancer of more than 50% (Ekbom *et al*, 1992, 1997). The authors attributed this effect to lower intrauterine oestrogen influence on foetal breast tissue in pre-eclampsia (Ekbom *et al*, 1992, 1997). However, the results of two recent studies indicate that an alternative interpretation may be relevant, since α-foetoprotein (AFP), a glycoprotein produced by the foetal liver and yolk sac, also has anti-oestrogenic properties (Speroff *et al*, 1983). Thus, a prospective study in Denmark has shown that high serum concentrations of maternal α-foetoprotein in pregnancy were related to lower risk of maternal breast cancer during follow-up (Melbye *et al*, 2000). An inverse association between third trimester levels of AFP and subsequent risk of breast cancer has also been reported by an American study (Richardson *et al*, 1998).

AFP enters the maternal circulation through the placenta and reaches its peak concentrations at the beginning of the third trimester (Speroff *et al*, 1983). In animal models, AFP can inhibit growth of oestrogen dependent mammary carcinomas *in vivo* (Jacobson *et al*, 1990; Bennett *et al*, 1998) and recently, it has been shown that human AFP peptides may bind the oestrogen receptor and suppress breast cancer cell growth (Vakharia and Mizejewski, 2000).

We have hypothesized that the anti-oestrogenic effect of pre-eclampsia could be mediated through high intrauterine levels of AFP, rather than low levels of oestradiol. We have further considered that an effect of severe pre-eclampsia could be related to reduced foetal growth (Ødegård *et al*, 2000; Roberts and Cooper, 2001). In a nested case–control study of pre-eclampsia in Norway, we have therefore compared oestradiol and AFP in umbilical cord plasma between cases of severe pre-eclampsia and control infants. We have also studied the relation between these two hormones in umbilical cord blood and infant birth weight.

## MATERIALS AND METHODS

Umbilical cord blood samples were collected in a prospective study of pregnancy outcome that took place from January 1993 to December 1995 at Rogaland Central Hospital in Stavanger, Norway. The maternity clinic at this hospital serves a region of approximately 239 000 inhabitants. Deliveries (12 804) took place during the study period. The Norwegian Medical Birth Registry records information on all deliveries that take place in the country (Lie *et al*, 1998), and we used this information to identify cases of pre-eclampsia and to select appropriate controls, as previously described (Ødegård *et al*, 2000).

From the Medical Birth Registry, we initially identified approximately 1300 cases with clinical information indicating possible pre-eclampsia or eclampsia. After verifying and supplementing this information with details from the hospital records, we identified 307 singleton pregnant women with definite pre-eclampsia. We used a previously described definition of pre-eclampsia in this study (CLASP Collaborative Group, 1994). Briefly, for pre-eclampsia to be diagnosed, persistent diastolic blood pressure of at least 90 mmHg had to develop after 20 weeks of gestation, and diastolic blood pressure had to increase by at least 25 mmHg. In addition, proteinuria had to be present, and cut-off was defined as 0.3 mg l^−1^ (semiquantitative dipstick 1+) in at least one urine sample after 20 weeks of gestation without simultaneous urinary infection.

Pre-eclampsia was classified as severe (*n*=66) if diastolic blood pressure increased to at least 110 mmHg, along with proteinuria 3+ on dipstick, or at least 500 mg per 24  h. Cases with eclampsia and suspected HELLP (haemolysis elevated liver enzymes, low platelets) syndrome were regarded interchangeable with severe pre-eclampsia. Cord plasma was analysed for all 66 cases of severe pre-eclampsia.

For comparison, women without pre-eclampsia were selected from the cohort of women who gave birth at Rogaland Central Hospital, as previously described (Ødegård *et al*, 2000). Among 619 control women, cord blood was available for analysis from 609. Information on baseline data was obtained at the first maternal visit at around 12 weeks of pregnancy. All infant data were compiled from hospital records.

Blood samples were collected after delivery from the placental side of the umbilical cord in syringes containing heparin, and chilled to 4°C up to 60 h before being centrifuged at 3000 r.p.m. for 15 min. Plasma was stored at −80°C until analysed.

Birth weight was standardised as the ratio between the observed and expected birth weight, the latter being adjusted for sex and gestational age at birth. We used standards of expected birth weights derived from the results of weight curves based on ultrasonographic measurements in a large Scandinavian population (Marsal *et al*, 1996). Gestational age at birth was calculated from routine ultrasonographic measurements at 18 weeks of gestation. Small-for-Gestational-Age (SGA) was defined as an observed birth weight two standard deviations or more below the expected, which corresponds to a ratio lower than 0.76, or to a birth weight reduction of approximately 840 g for a term infant.

We measured oestradiol and AFP using commercially available fluoroimmunoassays (Wallac Oy, Turku, Finland). Single samples were analysed, and procedures suggested by the manufacturer were followed. Oestradiol and AFP were detected in all plasma samples. In both assays, the intra-assay coefficients of variation at high and low levels were always less than 9%.

Oestradiol and AFP had positively skewed distributions, and we used Mann–Whitney *U*-test for comparisons between the groups. Differences between proportions were assessed by chi-square tests. The standardised birth weight was divided into four categories: <0.76 corresponds to a strict definition of small for gestational age (SGA), and 0.76–0.89 is a broad category of relatively small infants. The category 0.90 to 1.09 includes infants with appropriate weight for their gestation, and the category >1.09 includes large babies. For each level of birth weight, we estimated values of oestradiol and AFP in the pre-eclampsia group and in controls. In multiple linear regression analyses, we assessed whether the contribution to AFP or oestradiol differed between cases of severe pre-eclampsia and controls, after adjustment for length of gestation, birth weight and offspring sex. All statistical analyses were calculated using the Statistical Package for the Social Sciences (SPSS), version 10.05 (SPSS, Inc., Chicago, IL, USA).

## RESULTS

Table 1

**Table 1:**
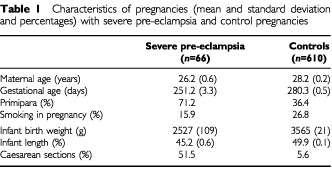
Characteristics of pregnancies (mean and standard deviation and percentages) with severe pre-eclampsia and control pregnancies

shows that in severe pre-eclampsia, mean maternal age was 26.2 *vs* 28.2 years among controls, whereas mean length of gestation was 251 and 280 days. In the pre-eclampsia group, 71% were primipara against 36.4% among controls. Infant birth weight was substantially lower after severe pre-eclampsia (mean: 2527 *vs* 3565 grams), as was infant length (mean: 45.2 *vs* 49.9 cm).

In Table 2

**Table 2:**
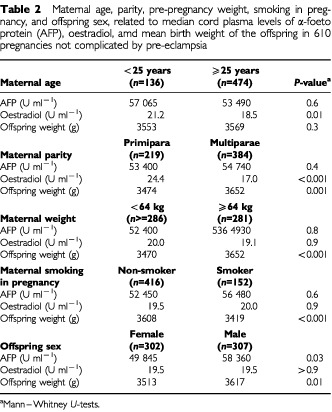
Maternal age, parity, pre-pregnancy weight, smoking in pregnancy, and offspring sex, related to median cord plasma levels of α-foeto protein (AFP), oestradiol, amd mean birth weight of the offspring in 610 pregnancies not complicated by pre-eclampsia

, we describe the control population according to categories of maternal factors and sex of the infant. We found higher oestradiol (*P*<0.03) in umbilical cord plasma in younger women, but no difference in AFP by maternal age. In primipara, cord plasma oestradiol was higher than in multiparae (*P*<0.001), and there was a consistent reduction in cord plasma oestradiol (*P* for trend <0.001) with increasing parity (Figure 1

**Figure 1:**
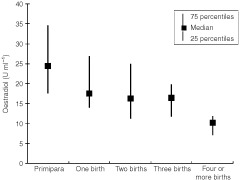
Oestradiol in umbilical cord plasma according to maternal parity.

). For AFP, we found no association with parity. There were no associations between pre-pregnancy weight and cord levels of oestradiol or AFP, and oestradiol and AFP did not significantly differ between smokers and non-smokers. Offspring birth weight was higher among multiparae than among primipara, it was positively associated with maternal pre-pregnancy weight, and birth weight was 188 g lower (*P*<0.001) if the mother reported smoking at the beginning of pregnancy. Birth weight was 106 g higher (*P*=0.01) in boys than girls. Oestradiol did not significantly differ by sex of the newborn, but AFP was higher in boys than in girls (*P*<0.02).

Table 3

**Table 3:**
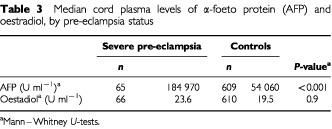
Median cord plasma levels of α-foeto protein (AFP) and oestradiol, by pre-eclampsia status

shows that median levels of cord plasma AFP were approximately three times higher in severe pre-eclampsia than in controls (*P*<0.001). In contrast, there was no difference in oestradiol levels between the groups. Across categories of standardised birth weight (Table 4

**Table 4:**
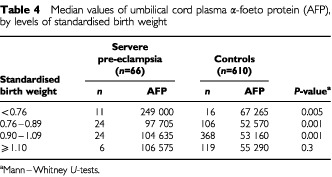
Median values of umbilical cord plasma α-foeto protein (AFP), by levels of standardised birth weight

), AFP was fairly uniform for both groups, but at each level of birth weight, the absolute value of AFP was approximately twice as high in the pre-eclampsia group as in controls. Adjustment for length of gestation (in days) attenuated the positive association between severe pre-eclampsia and AFP (Table 5

**Table 5:**
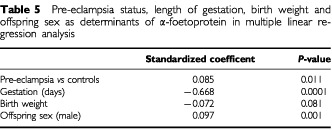
Pre-eclampsia status, length of gestation, birth weight and offspring sex as determinants of α-foetoprotein in multiple linear regression analysis

), but it remained statistically significant (*P*=0.01). For oestradiol (Table 6

**Table 6:**
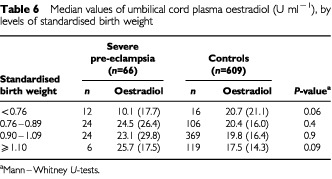
Median values of umbilical cord plasma oestradiol (U ml^−1^), by levels of standardised birth weight

), the pre-eclampsia group had slightly but not significantly higher cord levels than the corresponding controls at each level of birth weight, and further adjustment for gestational age did not substantially change this result (data not shown).

## DISCUSSION

Severe pre-eclampsia is characterised by abnormally shallow decidual invasion by the trophoblast, placental hypoxia, and reduced uteroplacental blood flow (Roberts and Cooper, 2001). We found that umbilical cord plasma AFP was substantially higher in severe pre-eclampsia compared to controls, whereas cord plasma oestradiol did not differ between the groups. Neither AFP nor oestradiol varied systematically with birth weight, but at each level of birth weight, AFP was consistently and significantly higher in severe pre-eclampsia than in controls.

To our knowledge, no previous study has used umbilical measurements of AFP and oestradiol to examine the association between pre-eclampsia and birth weight. Our study was performed within a population of nearly 13 000 consecutive births (Ødegård *et al*, 2000) for whom umbilical cord plasma was collected. The large number of pregnancies and a strict clinical definition of pre-eclampsia (CLASP Collaborative Group, 1994) allowed us to reliably distinguish between mild and severe disease. We have also benefited from the results of a longitudinal study of Scandinavian pregnancies (Marsal *et al*, 1996). By using repeated ultrasonographic scans, the objective of that study was to establish standards for foetal growth. Expected birth weights were derived from these standards, and dividing observed birth weight by the expected yielded standardised estimates for birth weight adjusted for gestational age at birth and offspring sex.

The results in the control population (Table 2) were in line with previous research in that lower birth weight was related to primiparity, maternal smoking, and to the newborn being of the female sex. This supports the validity of the novel results of our investigation. We found substantially higher cord levels of AFP in boys than girls, but no sex difference for oestradiol. Moreover, we found that cord levels of oestradiol were inversely related to maternal age and parity. This has been demonstrated previously in maternal blood (Panagiotopoulou *et al*, 1990), and our results show a similar relation for oestradiol measured in cord blood.

In uncomplicated pregnancies, α-foetoprotein reaches its maximum at the beginning of the third trimester, and drops thereafter towards term (Speroff *et al*, 1983). Cases of severe pre-eclampsia typically require that births will be therapeutically induced up to several weeks before term. Thus, collection of cord blood may coincide with a period in gestation when AFP levels would normally be higher than at term. Our results confirm that the association between severe pre-eclampsia and AFP was confounded by differences in gestational age. By taking these differences into account, the association was substantially attenuated, but remained statistically significant.

Recent studies have found that women who were born after pre-eclamptic pregnancies have a reduced risk of breast cancer in adult life (Ekbom *et al*, 1992, 1997; Sanderson *et al*, 1996). Several studies have also suggested that low birth weight may be associated with reduced breast cancer risk (Ekbom *et al*, 1992; Michels *et al*, 1996; Stavola *et al*, 2000). Since severe pre-eclampsia clearly reduces birth weight (Ødegård *et al*, 2000), one could speculate that the risk reduction linked to pre-eclampsia is mediated by restricted foetal growth. However, we found higher cord levels of AFP in the severe pre-eclampsia group than in controls, even after adjustment for birth weight and length of gestation. This finding may reinforce recent experimental evidence that AFP may be a factor with breast cancer inhibiting potential (Bennett *et al*, 1998, Vakharia and Mizejewski, 2000). In adulthood, two studies have recently related maternal blood levels of AFP during the second or third trimester among pregnant women to these women's subsequent risk of breast cancer (Richardson *et al*, 1998; Melbye *et al*, 2000). Both studies found that high pregnancy levels of AFP were associated with lower maternal breast cancer risk, and the authors attributed their findings to an anti-oestrogenic effect of AFP. Our findings may be important because they indicate that elevated AFP levels are associated with reduced breast cancer risk, not only among the pregnant women, but also among their female offspring.
